# Development and Validation of a Prediction Tool for Reoffending Risk in Domestic Violence

**DOI:** 10.1001/jamanetworkopen.2023.25494

**Published:** 2023-07-26

**Authors:** Rongqin Yu, Yasmina Molero, Paul Lichtenstein, Henrik Larsson, Lewis Prescott-Mayling, Louise M. Howard, Seena Fazel

**Affiliations:** 1 Department of Psychiatry, Warneford Hospital, University of Oxford, Oxford, United Kingdom; 2 Department of Clinical Neuroscience, Karolinska Institutet, Stockholm, Sweden; 3 Department of Medical Epidemiology and Biostatistics, Karolinska Institutet, Stockholm, Sweden; 4 Thames Valley Police, Kidlington, United Kingdom; 5 Department of Women & Children’s Health, King’s College London, London, United Kingdom

## Abstract

**Importance:**

Current risk assessment tools for domestic violence against family members were developed with small and selected samples, have low accuracy with few external validations, and do not report key performance measures.

**Objective:**

To develop new tools to assess risk of reoffending among individuals who have perpetrated domestic violence.

**Design, Setting, and Participants:**

This prognostic study investigated a national cohort of all individuals arrested for domestic violence between 1998 and 2013 in Sweden using information from multiple national registers, including National Crime Register, National Patient Register, Longitudinal Integrated Database for Health Insurance and Labour Market Studies Register, and Multi-Generation Register. Data were analyzed from August 2022 to June 2023.

**Exposure:**

Arrest for domestic violence.

**Main Outcomes and Measures:**

Prediction models were developed for 3 reoffending outcomes after arrest for domestic violence: conviction of a new violent crime (including domestic violence), conviction of any new crime, and rearrest for domestic violence at 1 year, 3 years, and 5 years. The prediction models were created using sociodemographic factors, criminological factors, and mental health status–related factors, linking data from multiple population-based longitudinal registers. Cox proportional hazard multivariable regression was used to develop prediction models and validate them in external samples. Key performance measures, including discrimination at prespecified cutoffs and calibration statistics, were investigated.

**Results:**

The cohort included 27 456 individuals (mean [SD] age, 39.4 [11.6] years; 24 804 men [90.3%]) arrested for domestic violence, of whom 4222 (15.4%) reoffended and were convicted for a new violent crime during a mean (SD) follow-up of 26.5 (27.0) months, 9010 (32.8%) reoffended and were convicted for a new crime (mean [SD] follow-up, 22.4 [25.1] months), and 2080 (7.6%) were rearrested for domestic violence (mean [SD] follow-up, 25.7 [30.6] months). Prediction models were developed with sociodemographic, criminological, and mental health factors and showed good measures of discrimination and calibration for violent reoffending and any reoffending. The area under the receiver operating characteristic curve (AUC) for risk of violent reoffending was 0.75 (95% CI, 0.74-0.76) at 1 year, 0.76 (95% CI, 0.75-0.77) at 3 years, and 0.76 (95% CI, 0.75-0.77) 5 years. The AUC for risk of any reoffending was 0.76 (95% CI, 0.75-0.77) at 1 year and at 3 years and 0.76 (95% CI, 0.75-0.76) at 5 years. The model for domestic violence reoffending showed modest discrimination (C index, 0.63; 95% CI, 0.61-0.65) and good calibration. The validation models showed discrimination and calibration performance similar to those of derivation models for all 3 reoffending outcomes. The prediction models have been translated into 3 simple online risk calculators that are freely available to use.

**Conclusions and Relevance:**

This prognostic study developed scalable, evidence-based prediction tools that could support decision-making in criminal justice systems, particularly at the arrest stage when identifying those at higher risk of reoffending and screening out individuals at low risk of reoffending. Furthermore, these tools can enhance treatment allocation by enabling criminal justice services to focus on modifiable risk factors identified in the tools for individuals at high risk of reoffending.

## Key Points

**Question:**

Is it possible to develop scalable and accurate prediction models for calculating risk of reoffending among individuals arrested for domestic violence?

**Findings:**

This prognostic study used data from a population-based cohort of 27 456 individuals arrested in Sweden for domestic violence to develop risk prediction models for violent reoffending, any reoffending, and domestic violence reoffending. The tools had good discrimination and calibration for violent reoffending and any reoffending and modest discrimination for domestic violence reoffending.

**Meaning:**

These findings suggest that risk prediction tools could be used to screen out individuals who are at low risk of reoffending and identify individuals at high risk of reoffending, which may assist with treatment allocation for health and justice services and stratify those who could benefit most from such services.

## Teaser

This prognostic study uses data from a population-based cohort of individuals arrested for domestic violence in Sweden to develop risk prediction models for domestic violence reoffense.

## Introduction

Domestic violence is highly prevalent globally. In the vast majority of cases, it is experienced by women or girls and is perpetrated by men.^1^ On average, approximately one-third of women and girls experience domestic violence, with prevalence rates ranging from 16% in Central Europe, to 25% in North America, and 44% in Central sub-Saharan Africa.^2^ There have been more studies of violence against men in recent years, reporting varying lifetime rates between 3% and 20%.^3^ Globally, 1 in 5 children have experienced maltreatment.^4^ Reoffending is common; some studies have reported that approximately one-half of survivors of domestic violence report reoccurence of domestic violence within 12 months,^5^ and one-half of individuals who have perpetrated domestic violence commit a new episode of general violence within 3 months.^6^ Experiencing domestic violence is associated with a range of long-term adverse outcomes, including premature mortality, self-harm, depression, anxiety, and posttraumatic stress disorder.^1,7,8,9^ Thus, reducing reoffending is a major focus for the criminal justice system, health care services, and public policy. As part of any violence prevention strategy, accurate risk assessment can assist with targeting interventions and law enforcement measures, identifying modifiable risk factors and linking them to treatment, and potentially improving resource allocation. Efficiently allocating resources has becoming increasingly important with underfunded public services in many countries.

Risk assessment tools for individuals who have perpetrated domestic violence have mostly used a structured judgment approach by clinicians and practitioners, whereby a short list of unweighted factors forms the basis of a final professional view of high, medium, or low risk. A minority of tools use an actuarial approach, whereby risk factors are combined into a model that gives a probability score. Several systematic reviews^10,11,12^ have reported that current tools have low to moderate predictive validity. In a recent review^12^ of 50 independent studies examining 39 tools, overall discriminative accuracy, as reported in area under the receiver operating characteristic curves (AUCs), was 0.60 after adjusting for bias. This poor performance is partly due to development in small and selected samples, with an average sample of fewer than 300 individuals.^13^ In addition, actuarial (or probabilistic) tools outperform structured clinical tools for domestic violence (AUC, 0.66 vs 0.58).^12^ However, current actuarial tools are based on dated methods without testing in multivariable models. They are susceptible to bias and have low accuracy in prediction, especially for reoffending with severe outcomes,^14,15^ although the tools often lack a clear definition of the outcome being predicted.^16^ Risk categories are inconsistent within and between police departments, probation officers, and third-sector practitioners,^17^ partly owing to the use of broad risk categories. Furthermore, current tools are typically lengthy, require costly training, and are resource intensive. In addition, they focus on survivors of domestic violence^18^ and are not shown to reduce reoffending.^19^ Rather, they need to be complemented by tools assessing risk in individuals who have perpetrated domestic violence.^20^ The lack of a valid tool to screen for individuals who have perpetrated domestic violence is a barrier to early identification and referral and appropriate law enforcement and risk management.

In this study, we aimed to develop risk prediction models for reoffending outcomes in individuals arrested for domestic violence. We have used data from a large nationwide population-based cohort of all people arrested for domestic violence in Sweden over a period of 16 years.

## Methods

This prognostic study was approved by the regional ethics committee at Karolinska Institutet and did not require informed consent because the data were deidentified, population wide, and register based, and, thus, it was not feasible to collect on this scale. This study complies with the Transparent Reporting of a Multivariable Prediction Model for Individual Prognosis or Diagnosis (TRIPOD) reporting guideline.^21^ We identified a national cohort of all individuals aged 15 years (ie, the age of criminal responsibility in Sweden) or older arrested for domestic violence between 1998 and 2013 in Sweden. We included an arrest cohort because our aim was to develop a tool to assist police in improving decision-making in law enforcement approaches and because domestic violence is underreported and has low conviction rates. We, therefore, focused on arrested individuals to capture more cases. Domestic violence is legally defined as acts of repeated violence, threats, harassment, and/or repeated violation against a person’s integrity, including coercive control, in close relationships or domestic contexts, where the person toward whom violence is directed is a partner, ex-partner, child, parent, or sibling of the offender. To ensure selection of a representative sample, we followed up individuals from the arrest date of 1 randomly selected domestic violence arrest from 1998 to 2013 to the first future reoffending date, death, or to the end of the study (December 31, 2013). We obtained information on crimes from the National Register for Suspected Offences, which includes all arrests in Sweden.^22^

### Measurement of Risk Factors

We linked data from national prospective registers by using the unique personal identification number carried by all citizens of Sweden; only 0.05% of all registered arrests had incomplete personal identification numbers. We examined risk factors from several domains, including sociodemographic factors, criminological factors, and mental health status–related factors, on the basis of prior empirical and conceptual models.^1,23,24,25^ Sociodemographic factors included age, sex, education, single status, and employment status at the time of arrest; these data were collected from the Longitudinal Integrated Database for Health Insurance and Labour Market Studies Register.^26^

Criminological factors were prior arrest for domestic violence before the index domestic violence incident, previous nondomestic violence offending, previous nonviolent offending, and previous imprisonment. Through linkage with the Multi-Generation Register at Statistics Sweden,^27^ we included parent crime history, such as parental violence.^28^ Parental violence was defined as any violent conviction of either parent before the index domestic violence arrest. Data were collected from the National Crime Register.^22^

Information about mental health and prior experience of violence were derived from health care data in the Swedish National Patient Register^29^ (see eTable 1 in Supplement 1 for diagnosis codes). Mental health status included any alcohol use disorder, drug use disorders, and any other mental disorders. We used any other mental disorders as a general risk factor, instead of including each specific type of mental disorder, because criminal justice services including those in corrections, which may validate and use such a model, do not typically have valid data on individual mental health diagnoses. In addition, we examined previous experience of violence.

### Measurement of Outcomes

Primary outcomes were first violent (including domestic violence) reoffending after the index domestic violence arrest at 1, 3, and 5 years. We examined both short-term and long-term reoffending outcomes to meet the needs of different service processes. Violent reoffending was defined as any violent offense that resulted in conviction occurring after the index domestic violent offense. Violent crimes included homicide, assault, robbery, arson, any sexual offense, illegal threats, domestic violence, or intimidation. We also included any reoffending (including violent and nonviolent crimes that led to conviction) and domestic violence reoffense that resulted in rearrest as secondary outcomes. Because each offense has a unique code and occurrence dates recorded in the crime registers, we were able to identify whether the conviction was the outcome of a new offense independently from the index domestic violence arrest.

### Statistical Analysis

We conducted a multivariable Cox proportional hazard regression to examine the association of sociodemographic, criminological, and mental health–related factors with reoffending outcomes, while accounting for time to event. We did not take a nonparametric approach because there were no strong theoretical and empirical bases for nonlinear associations of the included factors with reoffending outcomes. In addition, most of the factors were binary data, and nonlinearity could not be tested. For individuals imprisoned between the index arrest date and the date of reoffense, the length of imprisonment was deducted when calculating time at risk. We separated the samples into a derivation sample and a validation sample by adopting a stratified random selection approach based on the residential region of the individual (ie, urban areas of major cities, suburbs of major cities, counties with medium-sized populations, and counties with small populations) at the year of the arrest for domestic violence. This method is superior to randomly splitting the sample and is recommended by some experts.^30^ We used the derivation sample to generate models for predicting reoffending outcomes after arrest for the index domestic violence and the validation sample to test the predictive performance of models from the derivation sample in an external sample.

To calculate missing values, we conducted 20 imputations, with regression models that used data from other factors and the studied outcome, using the Nelson-Aalen cumulative hazard function.^30^ The final model included all selected variables that retained significance (2-sided *P* < .05) in multivariable analyses.

To examine the predictive ability of the identified final model, we tested both discrimination and calibration. We used the Harrell C index as an overall measure of discrimination, defined as the ability of the model to differentiate between individuals with and without reoffending outcomes during follow-up. The C index ranges from 0.5 to 1.0, with 1.0 representing perfect discrimination.^31^ We calculated the AUCs for outcomes and estimated the absolute predicted probabilities according to the regression model coefficients and baseline survivor function within the follow-up periods. Different AUC values can indicate varying levels of discrimination (≥0.80, excellent; 0.70 to <0.80, good; 0.60 to <0.70, fair; <0.60, poor). We reported sensitivity, specificity, positive predictive value, and negative predictive value and 95% CIs using evidence-based^32^ and prespecified thresholds (5%, 10%, 15%, and 20%) for the presence or absence of reoffending outcomes.

We examined calibration, which indicates how close the predicted risks were to the observed risks, by plotting these risks against each other. We also calculated Brier scores,^33^ the mean quadratic difference between the predicted probability and the observed binary outcome. The Brier score ranges from 0 to 1, with lower scores indicating better calibration. We conducted analyses using STATA statistical software version 17 (StataCorp) from August 2022 to June 2023. Finally, we used the final models for the different outcomes to develop 3 online risk calculators^34^ that provide probability scores and risk category.

## Results

Table 1 shows the baseline characteristics of a cohort of 27 456 individuals (mean [SD] age, 39.4 [11.6] years; 24 804 men [90.3%]) arrested for domestic violence between 1998 and 2013, including sociodemographic, criminological, and mental health–related factors. In the overall cohort, during a mean (SD) follow-up of 26.5 (27.0) months, reoffense was perpetrated by 4222 individuals (15.4%) who were subsequently convicted of an incident of violent crime, and 9010 individuals (32.8%; mean [SD] follow-up 22.4 [25.1] months) who were convicted of a new incident of any new crime, both of which were not linked to the initial index arrest for domestic violence. In addition, reoffense was perpetrated by 2080 individuals (7.6%; mean [SD] follow-up 25.7 [30.6] months) who were rearrested for domestic violence. The derivation sample (22 230 individuals) and the external validation sample (5226 individuals) had similar baseline characteristics and reoffending rates across follow-up periods (Table 1 and Table 2).

**Table 1.** Baseline Characteristics of Individuals Arrested for Domestic Violence

Characteristics	Individuals, No. (%) (N = 27 456)	
	Derivation sample (n = 22 230)	Validation sample (n = 5226)
Age, mean (SD) [range], y	39.5 (11.6) [15.0-93.0]	38.9 (11.6) [15.0-87.0]
Sex		
Male	20 036 (90.1)	4768 (91.2)
Female	2194 (9.9)	458 (8.8)
Education		
Secondary	7234 (34.2)	1803 (35.9)
Upper-secondary	10 172 (48.03)	2469 (49.1)
Postsecondary	3774 (17.8)	754 (15.0)
Single status^a^		
Yes	14 048 (64.2)	3372 (64.9)
No	7840 (35.8)	1825 (35.1)
Unemployed		
Yes	10 819 (49.4)	2685 (51.7)
No	11 069 (50.6)	2512 (48.3)
Alcohol use disorder^b^		
Yes	3282 (14.8)	729 (14.0)
No	18 948 (85.2)	4497 (86.1)
Drug use disorder^b^		
Yes	2136 (9.6)	555 (10.6)
No	20 094 (90.4)	4671 (89.4)
Any mental disorder		
Yes	6156 (27.7)	1653 (31.6)
No	16 074 (72.3)	3753 (68.4)
Previous domestic violence		
Yes	1712 (7.7)	361 (6.9)
No	20 518 (92.3)	4865 (93.1)
Previous nondomestic violence		
Yes	11 526 (51.9)	2720 (52.1)
No	10 704 (48.2)	2506 (47.9)
Previous nonviolent offense		
Yes	4524 (20.4)	1017 (19.5)
No	17 706 (79.7)	4209 (80.5)
Previous imprisonment		
Yes	2714 (12.2)	670 (12.8)
No	19 516 (87.8)	4556 (87.2)
Previous recipient of violence		
Yes	1929 (8.7)	411 (7.9)
No	20 301 (91.3)	4815 (92.1)
Parental violence		
Yes	2078 (9.4)	532 (10.2)
No	20 152 (90.7)	4694 (89.8)

^a^ Refers to status at time of arrest for domestic violence.

^b^ Refers to clinical diagnosis before arrest for domestic violence.

**Table 2.** Rates of Violent Reoffending, Any Reoffending, and Domestic Violence Reoffending

Type of reoffending and time period, y	Individuals, No. (%) (N = 27 456)	
	Derivation sample (n = 22 230)	Validation sample (n=5226)
Violent offense	3322 (15.0)	900 (17.2)
1	1301 (5.9)	390 (7.5)
3	2443 (11.0)	686 (13.1)
5	2930 (13.2)	796 (15.2)
Any offense	7218 (32.5)	1792 (34.3)
1	3385 (15.2)	908 (17.4)
3	5737 (25.8)	1442 (27.6)
5	6564 (29.5)	1639 (31.4)
Domestic violence offense	1690 (7.6)	390 (7.5)
1	795 (3.6)	177 (3.4)
3	1289 (5.8)	289 (5.5)
5	1479 (6.7)	336 (6.4)

**Figure 1.**
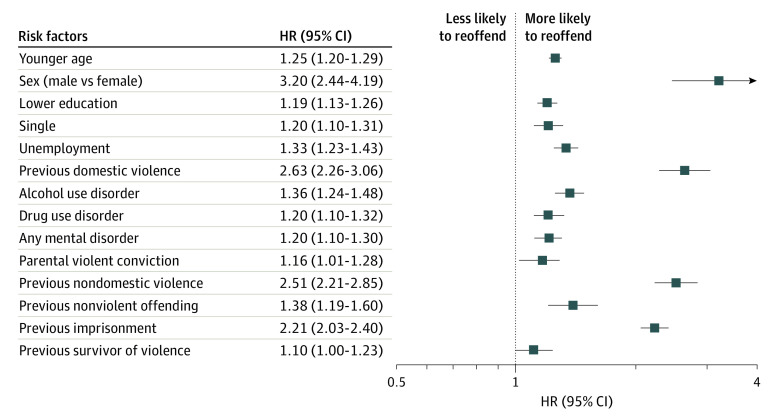
Factors Included in the Final Model for Prediction of Violent Reoffending The forest plot shows the hazard ratios (HRs) for various characteristics associated with violent reoffending. Boxes denote HRs, and lines denote 95% CIs. Younger age refers to the effect per 10 years of age. Unemployment and single status refer to status at time of arrest of domestic violence. HRs were for the whole study period with a follow-up of 26.5 months.

**Figure 2.**
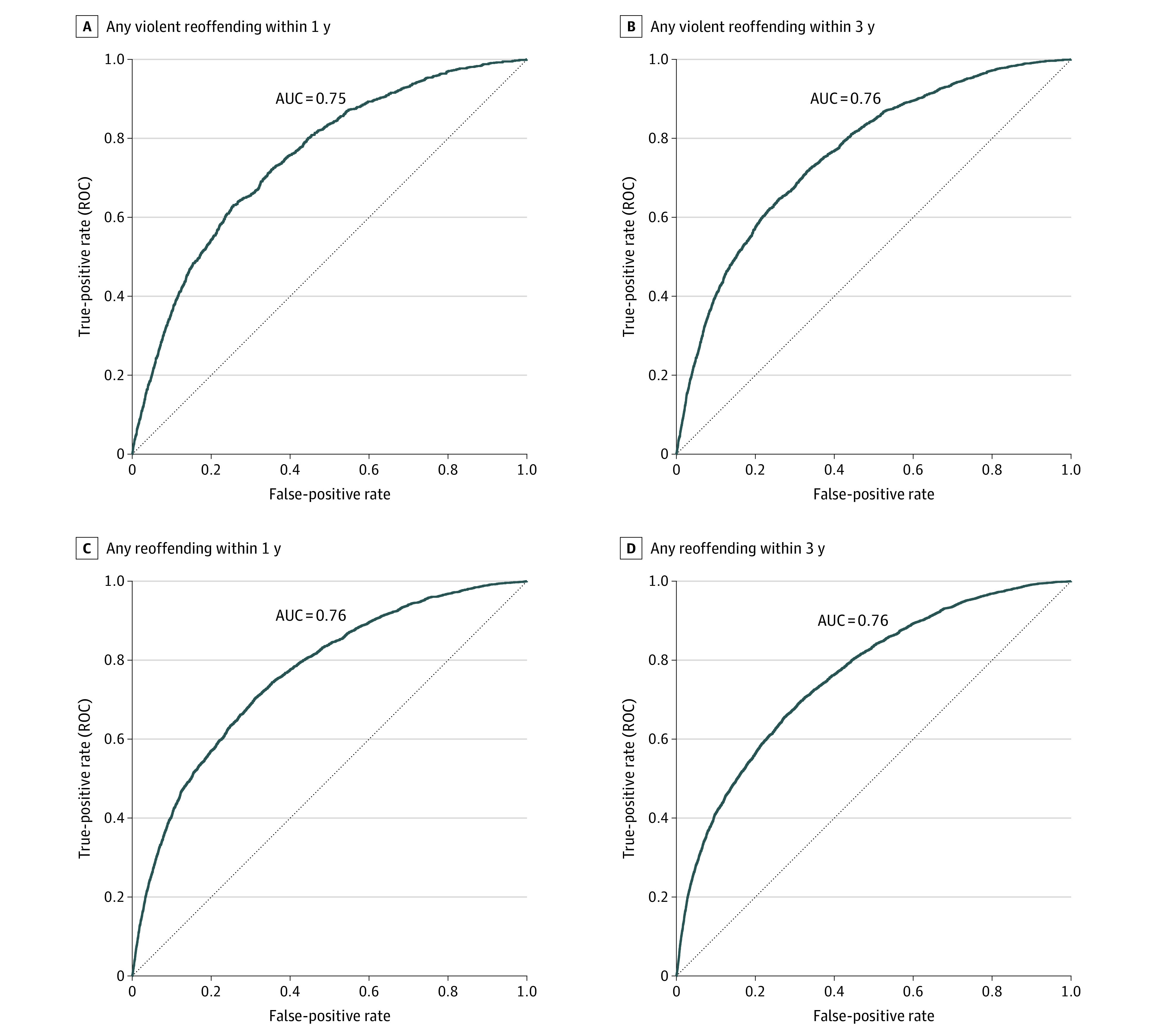
Model Discrimination Shown by Receiver Operating Characteristic Curves for Reoffending in the Derivation Sample Figure shows receiver operating curves for any violent reoffending within 1 year (A), any violent reoffending within 3 years (B), any reoffending within 1 year (C), and any reoffending within 3 years (D). AUC indicates area under the receiver operating characteristic curve.

**Figure 3.**
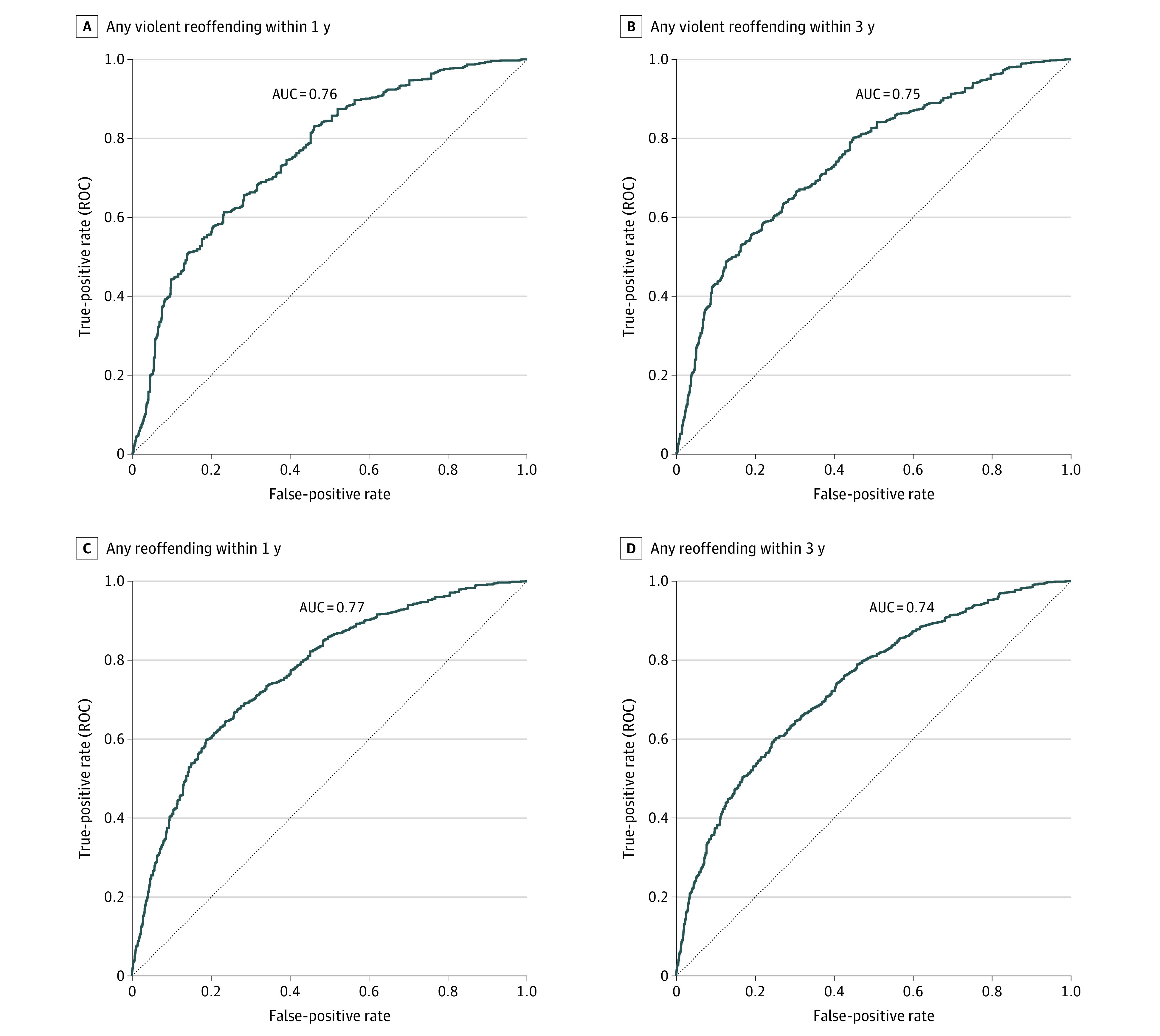
Model Discrimination Shown by Receiver Operating Characteristics Curves for Reoffending in the Validation Sample Figure shows receiver operating curves in the validation sample for any violent reoffending within 1 year (A), any violent reoffending within 3 years (B), any reoffending within 1 year (C), and any reoffending within 3 years (D). AUC indicates area under the receiver operating characteristic curve.

The final model for violent reoffending included the following factors: younger age, sex (male vs female), lower education level, single status, previous domestic violence offense, previous nondomestic violence offense, previous nonviolent offense, prior imprisonment, a diagnosis of alcohol use disorder, a diagnosis of drug use disorder, parental violence, and previous recipient of violence. eTable 2 in Supplement 1 shows model coefficients, and Figure 1 shows hazard ratios (HRs). The C index was 0.74, indicating good overall discrimination. The derivation model showed good discrimination for violent reoffending within 1 year (AUC, 0.75; 95% CI, 0.74-0.76), 3 years (AUC, 0.76; 95% CI, 0.75-0.77), and 5 years (AUC, 0.76; 95% CI, 0.75-0.77) (Figure 2A, Figure 2B, Figure 3, and eFigure 1 in Supplement 1). Importantly, the external validation also reported good discrimination for violent reoffending (AUC, 0.74 [95% CI, 0.72-0.75] to 0.76 [95% CI, 0.74-0.77]) (Figure 3A, Figure 3B, and eFigure 2 in Supplement 1) for all 3 follow-up periods. Other discrimination measures, including sensitivity, specificity, positive predictive value, and negative predictive value for prespecified cutoffs (5%, 10%, 15%, and 20%), are presented in eTable 3 in Supplement 1.

The model showed good calibration for violent reoffending predictions (eFigure 3 in Supplement 1). The expected-to-observed risk ratios ranged from 1.04 to 1.22 (where 1 would indicate perfect calibration). The Brier score was 0.05 for violent reoffending within 1 year, 0.09 for within 3 years, and 0.10 for within 5 years after the index domestic violence arrest (Brier scores of 0 indicate perfect calibration). All Brier scores were lower than those obtained when using the mean predicted probability or using 0 (eTable 4 in Supplement 1). The external validation model showed similar discrimination and calibration performance (Figure 3A and Figure 3B).

When we investigated any reoffending as a secondary outcome, we included 14 risk factors in the final model (see eTable 5 in Supplement 1 for model coefficients and eFigure 4 in Supplement 1 for HRs). The derivation model showed good discrimination and calibration (eFigure 6 in Supplement 1). The C index was 0.72. AUCs were 0.76 (95% CI, 0.75-0.77) for 1 year and 3 years and 0.76 (95% CI, 0.75-0.76) for 5 years (Figure 2C, Figure 2D, and eFigure 1 in Supplement 1).

It is notable that the external validation reported good discrimination for any reoffending (AUCs ranged from 0.72 [95% CI, 0.71-0.73] to 0.77 [95% CI, 0.75-0.78]) (Figure 3C, Figure 3D, and eFigure 5 in Supplement 1) and good calibration, with low expected-to-observed risk ratios and Brier scores (eTable 4 in Supplement 1). Discrimination measures are reported in eTable 6 in Supplement 1.

In addition, we tested the model for predicting reoffending for domestic violence, which included 7 risk factors (see eTable 7 in Supplement 1 for model coefficients and eFigure 7 in Supplement 1 for HRs). The model showed moderate discrimination, with the C index ranging from 0.63 (95% CI, 0.61-0.65) to 0.65 (95% CI, 0.63-0.66) for reoffending outcomes within 1, 3, and 5 years (eFigure 8 in Supplement 1), and good calibration, with Brier scores ranging from 0.03 to 0.06 (eFigure 9 and eTable 4 in Supplement 1). Other discrimination measures are presented in eTable 8 in Supplement 1. We created 3 online calculators on the basis of the coefficients of the final models.^34^

## Discussion

This prognostic study examined a national cohort of 27 456 individuals arrested for domestic violence over 16 years in Sweden, and investigated the risk of violent reoffending, any reoffending, and domestic violence reoffending outcomes. Reoffending rates were 32.8% for conviction of any new crime, 15.4% for conviction of a new violent crime, and 7.6% for rearrest for a new episode of domestic violence, over a mean follow-up period of 22.4 to 26.5 months. We developed separate risk prediction models for these reoffending outcomes using national registers across sociodemographic, criminological, and mental health factors, which were based on predictors that were routinely collected. The models performed well in terms of discrimination for violent reoffending and any reoffending but less well for domestic violence reoffending. Calibration was good for all of these models.

Substance use disorders and other mental disorders were associated with an increased risk of reoffending. For both violent reoffending and any reoffending outcomes, alcohol and drug use disorders were important factors, and in the model for domestic violence reoffending, alcohol use disorder was associated with later recidivism. Previous research^35^ has indicated that addressing substance use disorders and other mental health disorders can lead to a reduction in reoffending. The newly developed tools could assist when making decisions on how to allocate treatment and the extent and frequency of supervision. For instance, if an individual is identified to be at high risk for reoffending with these new tools, in addition to usual criminal justice measures such as arrest, police could refer the individuals at risk to local health services and other services for treatment of alcohol or drug misuse problems. These are important decisions for resource-limited public services, where there will be many competing demands. The predictive accuracy for the 3 outcomes varied, with better performance for general violent reoffending and any reoffending than that for domestic violence reoffending (ie, AUCs ≥0.72 vs 0.63). This finding contrasts with a recent systematic review^12^ that found no differences, with AUCs for both outcomes ranging from 0.64 to 0.65. However, we have developed models with higher AUCs for any reoffending (C index, 0.72) and violent reoffending (C index, 0.74). This finding is similar to recently developed tools^36^ for individuals convicted of sexual violence reoffense, showing higher AUCs for general and violent recidivism than that for sexual crime. The lower predictive validity in domestic violence reoffending could partly be explained by the lack of relationship-based factors in the new tool because they are not routinely collected in health care and crime registers. Furthermore, exposure to maltreatment during childhood has consistently been associated with domestic violence^37^; this information was not available in our study.

A notable advantage of these tools is that the contribution of each factor was examined in multivariable regression models. The newly developed calculators provide both a probability score and prespecified risk category for users, instead of only subjective categorizations, which could introduce bias among practitioners. Adding a probability score to risk assessments will likely help standardize discussions between police officers, criminal justice agencies, third-sector practitioners, and health services. The tools are brief, including 14 items for both violent reoffending and any reoffending, and 7 items for domestic violence reoffending, and have been translated to simple and easy online calculators (OxDoV)^34^ that are freely available. Given the time constraints and limited resources within criminal justice, third-sector practitioners, and health services, scalable tools should play an important role.

To facilitate practical interpretation, we calculated sensitivity, specificity, positive predictive value, and negative predictive value using different cutoff scores. These cutoff scores can be used to inform decision-making for different contexts and processes. False-positive errors are problematic for individuals and their families.^38^ However, if the goal is to identify individuals for intervention (as health care may prioritize), false-positives can be tolerated, and when resources permit, more individuals could be included for follow-up and treatment. False-negative errors come with substantial costs to public safety. If the purpose of the assessment is to reduce reoffending, higher sensitivities will be prioritized and lower cutoff scores can be used. The newly developed tools for violent and any reoffending can assist in both identifying individuals at high risk of reoffending and screening out individuals at low risk of reoffending because they could achieve high sensitivity and specificity.

We included sociodemographic factors, such as sex, in the prediction models. The theoretical rationale for including sex is that domestic violence is predominantly characterized as sex-based violence. Although concerns exist regarding the potential for disparities when incorporating sociodemographic factors into algorithms,^39^ sex is an important factor, with the highest HR among the variables examined. Removing sex from the model would result in a substantial decrease in its predictive performance. The ethical considerations of various predictors need consideration, including different approaches to weighing the potential benefits and harms.^40^

### Strengths and Limitations

This study has several strengths. We developed tools with data from a large national cohort of all people arrested, allowing for more consistent and reliable model performance compared with previously small and selected samples. The definition of domestic violence used in Sweden is similar to that used in other high-income countries, such as Spain, Germany, Canada, and the US, because it includes a broad scope of acts, such as violence, harassment, threats, and coercive behaviors, which supports the prediction model’s generalizability, although the definition of predictors and outcomes will also need consideration.

In addition to discriminative validity, we reported calibration, whereas most related studies only report concurrent and construct validity. Furthermore, we validated the tool in an external sample. Unlike most currently used tools, the newly developed ones also provide risk probability scores instead of categorizing individuals into different levels of risk according to arbitrary cutoff scores. This approach provides more flexibility because practitioners can choose a combination of probability and categorical scores to assist their decision-making, depending on the specific setting.

Several limitations should also be noted. First, the rate of domestic violence reoffending at 7.6% underreports domestic violence, partly because most individuals who experienced domestic violence do not report to the police, and police and other criminal justice agencies might misclassify domestic violence as general violence.^41^ Therefore, our findings are specific to the more severe forms of domestic violence that lead to arrest and are most likely to be associated with adverse outcomes for survivors of domestic violence. In addition, to examine whether the developed tools could be used in a new setting or to predict a related outcome, such as gun violence in the US, validation studies are needed. Second, we could not examine the effect of certain factors that are associated with domestic violence perpetration, such as exposure to childhood maltreatment and relationship quality with family members,^1,25,42,43^ because of a lack of these data in the registries. Third, we used data from individuals arrested for domestic violence up to 2013, because more recent data are currently not available for research purposes. Although the magnitude of the associations of factors with outcomes is unlikely to have materially altered^44^ and the outcome rates remain similar,^45^ external validation of the model using more recent data is required. Fourth, we examined all types of domestic violence together as an outcome, because data were not available to separate domestic violence into different types. Factors associated with different types of domestic violence may vary, and they should be explored and clarified in future studies.

## Conclusions

In conclusion, by using linked national data for 16 years from a population-based cohort of individuals arrested for perpetration of domestic violence, we have developed 3 risk assessment tools to predict reoffending outcomes. These tools could be used to assist decision-making in criminal justice and mental health services and assist with prevention initiatives.

## Notes

Supplement 1. **eTable 1.** *ICD* Codes for Mental Health and Prior Violence Victimization Included as Risk Factors **eTable 2.** Parameters to Calculate Predicted Risk of Violent Reoffending Within 1, 3 and 5 Years After Arrest for Domestic Violence **eFigure 1.** Model Discrimination Shown by Receiver Operating Characteristics Curves for Conviction of New Violent and Any Crime Within 5 Years **eFigure 2.** Model Discrimination Shown by Receiver Operating Characteristics Curves for Conviction of New Violent Crime After Arrested for Domestic Violence **eTable 3.** Sensitivity, Specificity, Negative Predictive Value, and Positive Predictive Value by Different Cutoff Scores for Probability of Violent Reoffending **eFigure 3.** Model Calibration for Conviction of a New Violent Crime After Arrest for Domestic Violence **eTable 4.** Brier Scores for Outcomes at Different Follow-Up Periods **eTable 5.** Parameters to Calculate Predicted Risk of Any Reoffending Within 1, 3 and 5 Years After Arrest for Domestic Violence **eFigure 4.** Risk Factors Included in the Final Model for Prediction of Conviction of a New Crime **eFigure 5.** Model Discrimination Shown by Receiver Operating Characteristics Curves for Any Conviction of a New Crime **eFigure 6.** Model Calibration for Any Conviction of a New Crime **eTable 6.** Sensitivity, Specificity, Negative Predictive Value, and Positive Predictive Value by Different Cutoff Scores for Probability of Any Reoffending **eTable 7.** Parameters to Calculate Predicted Risk of Domestic Violence Reoffending After Arrest for Domestic Violence **eFigure 7.** Risk Factors Included in the Final Model for Prediction of Rearrest for Domestic Violence **eFigure 8.** Model Discrimination Shown by Receiver Operating Characteristics Curves for Rearrest for Domestic Violence **eFigure 9.** Model Calibration for Domestic Violence Reoffending **eTable 8.** Sensitivity, Specificity, Negative Predictive Value, and Positive Predictive Value by Different Cutoff Scores for Probability of Domestic Violence Reoffending

Supplement 2.
Data Sharing Statement
