# Supplementary material for: Development and Validation of a Prediction Tool for Reoffending Risk in Domestic Violence
Source: JAMA Netw Open. 2023 Jul 26;6(7):e2325494. doi: 10.1001/jamanetworkopen.2023.25494 (PMC10372708; doi:10.1001/jamanetworkopen.2023.25494)
Supplement: Supplement 1. — eTable 1. ICD Codes for Mental Health and Prior Violence Victimization Included as Risk Factors eTable 2. Parameters to Calculate Predicted Risk of Violent Reoffending Within 1, 3 and 5 Years After Arrest for Domestic Violence eFigure 1. Model Discrimination Shown by Receiver Operating Characteristics Curves for Conviction of New Violent and Any Crime Within 5 Years eFigure 2. Model Discrimination Shown by Receiver Operating Characteristics Curves for Conviction of New Violent Crime After Arrested for Domestic Violence eTable 3. Sensitivity, Specificity, Negative Predictive Value, and Positive Predictive Value by Different Cutoff Scores for Probability of Violent Reoffending eFigure 3. Model Calibration for Conviction of a New Violent Crime After Arrest for Domestic Violence eTable 4. Brier Scores for Outcomes at Different Follow-Up Periods eTable 5. Parameters to Calculate Predicted Risk of Any Reoffending Within 1, 3 and 5 Years After Arrest for Domestic Violence eFigure 4. Risk Factors Included in the Final Model for Prediction of Conviction of a New Crime eFigure 5. Model Discrimination Shown by Receiver Operating Characteristics Curves for Any Conviction of a New Crime eFigure 6. Model Calibration for Any Conviction of a New Crime eTable 6. Sensitivity, Specificity, Negative Predictive Value, and Positive Predictive Value by Different Cutoff Scores for Probability of Any Reoffending eTable 7. Parameters to Calculate Predicted Risk of Domestic Violence Reoffending After Arrest for Domestic Violence eFigure 7. Risk Factors Included in the Final Model for Prediction of Rearrest for Domestic Violence eFigure 8. Model Discrimination Shown by Receiver Operating Characteristics Curves for Rearrest for Domestic Violence eFigure 9. Model Calibration for Domestic Violence Reoffending eTable 8. Sensitivity, Specificity, Negative Predictive Value, and Positive Predictive Value by Different Cutoff Scores for Probability of Domestic Violence Reoffending [file jamanetwopen-e2325494-s001.pdf]

Supplemental Online Content

Yu R, Molero Y, Lichtenstein P, et al. Development and validation of a prediction tool for reoffending risk in domestic violence. *JAMA Netw* Open. 2023;6(7):e2325494. doi:10.1001/jamanetworkopen.2023.25494

eTable 1. *ICD* Codes for Mental Health and Prior Violence Victimization Included as Risk Factors
eTable 2. Parameters to Calculate Predicted Risk of Violent Reoffending Within 1, 3 and 5 Years After Arrest for Domestic Violence
eFigure 1. Model Discrimination Shown by Receiver Operating Characteristics Curves for Conviction of New Violent and Any Crime Within 5 Years
eFigure 2. Model Discrimination Shown by Receiver Operating Characteristics Curves for Conviction of New Violent Crime After Arrested for Domestic Violence
eTable 3. Sensitivity, Specificity, Negative Predictive Value, and Positive Predictive Value by Different Cutoff Scores for Probability of Violent Reoffending
eFigure 3. Model Calibration for Conviction of a New Violent Crime After Arrest for Domestic Violence
eTable 4. Brier Scores for Outcomes at Different Follow-Up Periods
eTable 5. Parameters to Calculate Predicted Risk of Any Reoffending Within 1, 3 and 5 Years After Arrest for Domestic Violence
eFigure 4. Risk Factors Included in the Final Model for Prediction of Conviction of a New Crime
eFigure 5. Model Discrimination Shown by Receiver Operating Characteristics Curves for Any Conviction of a New Crime
eFigure 6. Model Calibration for Any Conviction of a New Crime
eTable 6. Sensitivity, Specificity, Negative Predictive Value, and Positive Predictive Value by Different Cutoff Scores for Probability of Any Reoffending
eTable 7. Parameters to Calculate Predicted Risk of Domestic Violence Reoffending After Arrest for Domestic Violence
eFigure 7. Risk Factors Included in the Final Model for Prediction of Rearrest for Domestic Violence
eFigure 8. Model Discrimination Shown by Receiver Operating Characteristics Curves for Rearrest for Domestic Violence
eFigure 9. Model Calibration for Domestic Violence Reoffending
eTable 8. Sensitivity, Specificity, Negative Predictive Value, and Positive Predictive Value by Different Cutoff Scores for Probability of Domestic Violence Reoffending

This supplemental material has been provided by the authors to give readers additional information about their work.

© 2023 Yu R et al. *JAMA Network Open.*

eTable 1. *ICD* codes for mental health and prior violence victimization included as risk factors.

**Mental health and prior violence victimization**	**ICD codes**
Alcohol use disorders	ICD-8: 291, 303<br>ICD-9: 291, 303, 305A<br>ICD-10: F10
Drug use disorders	ICD-8: 304<br>ICD-9: 292, 304, 305 (ex. A)<br>ICD-10: F11-F19
Any other mental disorders (excluding alcohol and drug use disorders)	ICD-8: 290-315;<br>ICD-9: 290-319; ICD-10: F00-F99, excluding codes for alcohol and drug use disorders
Previous violent victimization	ICD-9: E960–E969; ICD-10: X85–Y09

© 2023 Yu R et al. *JAMA Network Open.*

eTable 2. Parameters to calculate predicted risk of violent reoffending within 1, 3 and 5 years after arrest for domestic violence

Risk factors	Beta	LCI	UCI
Younger age band	.220413	.1858410	.254985
Gender (male vs. female)	1.160324	.8906512	1.429997
Education			
Secondary	0	0	0
Upper-Secondary	-.130387	-.2056981	-.0550759
Post-Secondary	-.4089439	-.5515529	-.2663349
Single	.1853120	.0999844	.2706396
Unemployment	.2909272	.2150476	.3668068
Previous domestic violence	.9555379	.8032894	1.107786
Alcohol use disorder	.3005242	.2100445	.3910040
Drug use disorder	.1818258	.0832849	.2803666
Any mental disorder	.1634459	.0813655	.2455262
Previous non-domestic violence	.9116243	.7830923	1.040156
Previous non-violent offending	.3165099	.1709196	.4621002
Previous imprisonment	.7901411	.7056122	.8746700
Previous violence victimization	.0986350	-.0065498	.2038198
Parental violent conviction	.1447788	.0472451	.2423125

Note: LCI = Lower Confidence Interval; UCI = Upper Confidence Interval.
Unemployment and single status refer to status at time of arrest for domestic violence.

© 2023 Yu R et al. *JAMA Network Open.*

Derivation sample

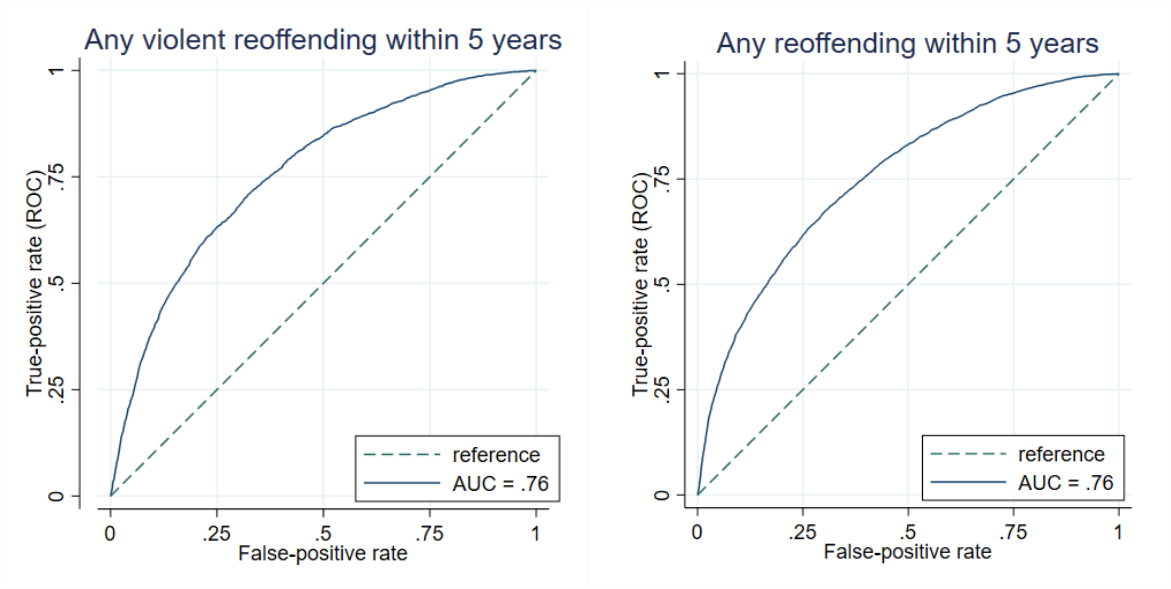

eFigure 1. Model discrimination shown by receiver operating characteristics curves for conviction of new violent and any crime within 5 years

Note: AUC = areas under the receiver operating characteristics curves.

© 2023 Yu R et al. *JAMA Network Open.*

Validation sample

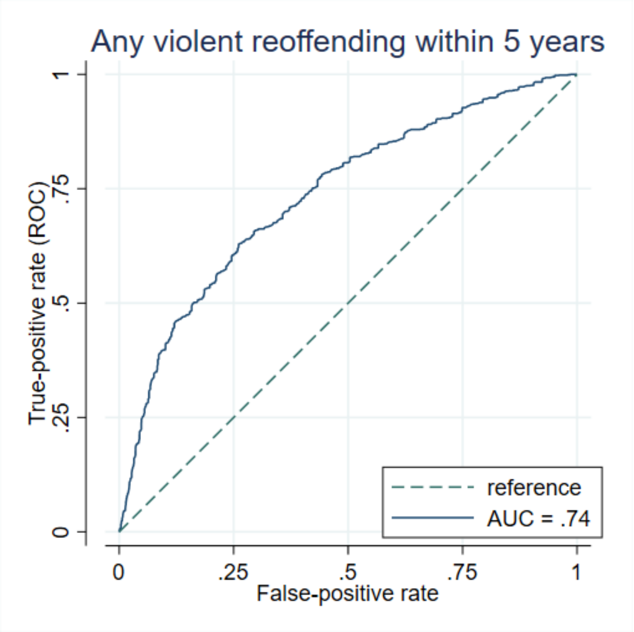

eFigure 2. Model discrimination shown by receiver operating characteristics curves for conviction of new violent crime after arrest for domestic violence

Note: AUC = areas under the receiver operating characteristics curve.

© 2023 Yu R et al. *JAMA Network Open.*

eTable 3. Sensitivity, specificity, negative predictive value, and positive predictive value by different cut-off scores for probability of violent reoffending

	Sensitivity (95%CI)		Specificity (95%CI)		PPV (95%CI)		NPV (95%CI)		Accuracy (95%CI)	
Cut-off										
Within 1 year (5.9%)										
5%	75.6	73.1, 77.9	60.4	59.7, 61.0	10.6	10.3, 10.9	97.6	97.3, 97.8	61.3	60.6, 61.9
10%	47.0	44.2, 49.7	85.3	84.8, 85.8	16.6	15.7, 17.5	96.3	96.1, 96.5	83.1	82.6, 83.5
15%	28.4	26.0, 31.0	92.6	92.2, 92.9	19.2	17.7, 20.7	95.4	95.3, 95.6	88.8	88.4, 89.2
20%	16.5	14.5, 18.6	96.1	95.9, 96.4	20.9	18.7, 23.3	94.9	94.8, 95.0	91.5	91.1, 91.8
Within 3 years (11.0%)										
5%	94.2	93.2, 95.1	28.8	28.2, 29.5	14.1	13.9, 14.2	97.6	97.2, 98.0	36.0	35.4, 36.7
10%	77.2	75.4, 78.8	59.4	58.7, 60.1	19.0	18.6, 19.4	95.5	95.1, 95.8	61.4	60.7, 62.0
15%	60.4	58.5, 62.4	77.7	77.1, 78.2	25.0	24.3, 25.8	94.1	93.8, 94.4	75.8	75.2, 76.3
20%	47.7	45.7, 49.7	86.4	86.0, 86.9	30.3	29.2, 31.5	93.1	92.8, 93.3	82.2	81.7, 82.7
Within 5 years (13.2%)										
5%	97.8	97.1, 98.3	17.9	17.4, 18.5	15.3	15.2, 15.4	98.1	97.6, 98.5	28.4	27.8, 29.0
10%	86.8	85.5, 88.0	46.9	46.2, 47.6	19.9	19.6, 20.2	95.9	95.5, 96.3	52.1	51.5, 52.8
15%	72.1	70.5, 73.7	66.3	65.6, 67.0	24.5	24.0, 25.1	94.0	93.7, 94.3	67.1	66.4, 67.7
20%	58.8	57.0, 60.6	79.0	78.4, 79.6	29.8	29.0, 30.7	92.7	92.4, 93.0	76.3	75.8, 76.9

Note: Data are given in percentages. 95%CI, 95% confidence interval; PPV, positive predictive value; NPV, negative predictive value.

© 2023 Yu R et al. *JAMA Network Open.*

Derivation sample

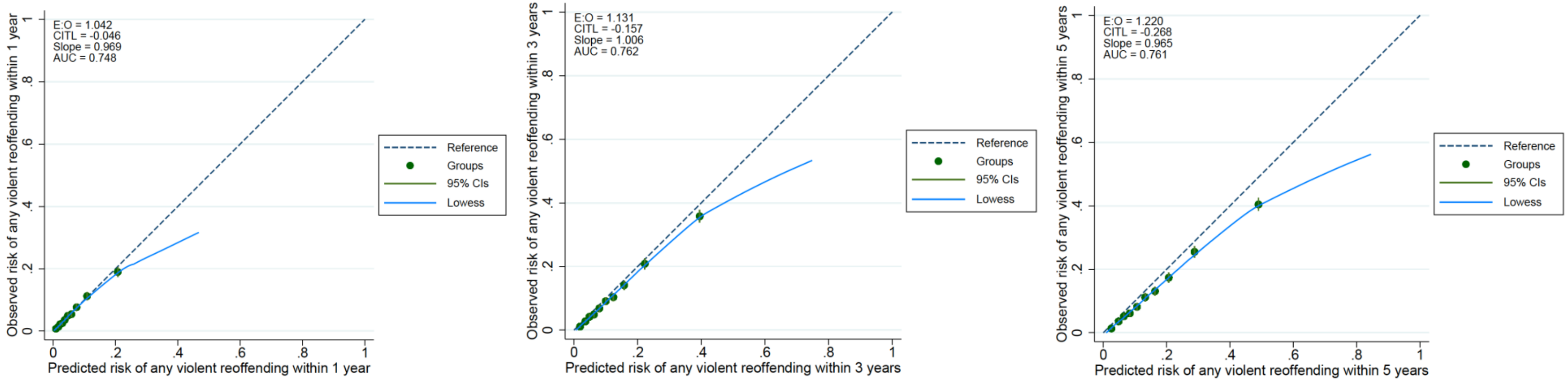

eFigure 3. Model calibration for conviction of a new violent crime after arrest for domestic violence

© 2023 Yu R et al. *JAMA Network Open.*

eTable 4. Brier scores for outcomes at different follow-up periods

	Brier scores								
	Within1 year			Within 3 years			Within 5 years		
	Predicted probability	Mean probability	Zero probability	Predicted probability	Mean probability	Zero probability	Predicted probability	Mean probability	Zero probability
Violent reoffending	.0523524	.0551053	.0585245	.0882511	.0980276	.1098965	.1030576	.1152705	.1318039
Any reoffending	.1120699	.1291324	.1522717	.1590365	.1927062	.2580747	.1756005	.2119123	.2952767
Domestic violence reoffending	.0342010	.0344852	.0357625	.0537859	.0546647	.0579847	.0609968	.0622506	.0665317

© 2023 Yu R et al. *JAMA Network Open.*

eTable 5. Parameters to calculate predicted risk of any reoffending within 1, 3 and 5 years after arrest for domestic violence

Risk factors	Beta	LCI	UCI
Younger age	.199829	.17706	.222597
Education			
Secondary	0	0	0
Upper-Secondary	-.1053170	-.1571728	-.0534612
Post-Secondary	-.3424799	-.4292359	-.2557239
Gender (male vs female)	.9894505	.8281887	1.150712
Victim gender (female vs male)	.1204480	.0439283	.1969677
Single	.1787854	.1228149	.2347558
Unemployed	.3419535	.2915357	.3923712
Alcohol use disorder	.1488652	.0860632	.2116672
Drug use disorder	.3365078	.2671643	.4058513
Previous domestic violence	.7311871	.6314382	.830936
Previous non-domestic violence	.8235980	.7438294	.9033665
Previous non-violent offending	.6811361	.594876	.7673962
Previous imprisonment	.8484444	.7862553	.9106334
Previous violent victimization	.1614818	.0872972	.2356664
Parental any offending	.1517112	.1008475	.2025749

Note: LCI = Lower Confidence Interval; UCI = Upper Confidence Interval.
Unemployment refers to status at time of arrest for domestic violence.

© 2023 Yu R et al. *JAMA Network Open.*

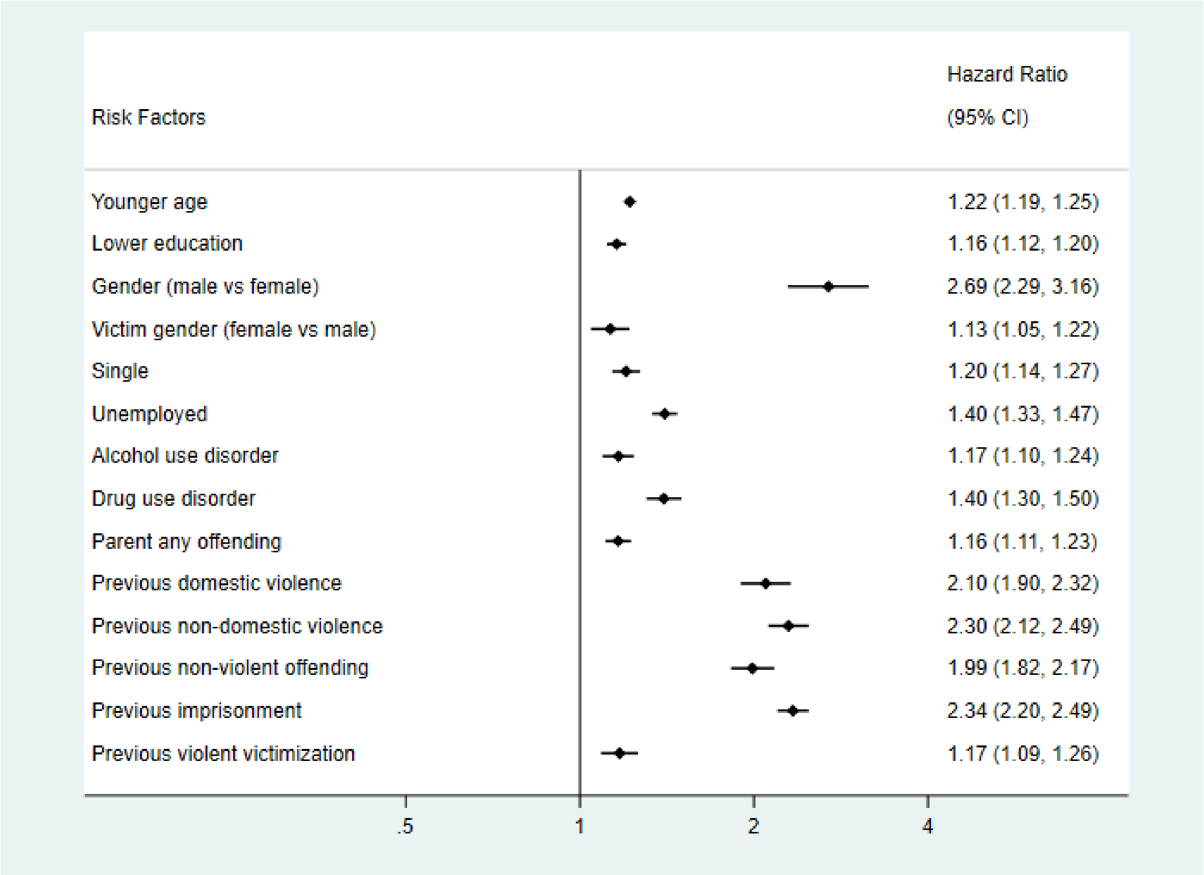

eFigure 4. Risk factors included in the final model for prediction of conviction of a new crime

Note: Younger age refers to the effect per 10 years of age. Unemployment refers to status at time of arrest for domestic violence. Hazard ratios were for the whole study period with a mean follow-up period of 22.4 months.

© 2023 Yu R et al. *JAMA Network Open.*

Validation sample

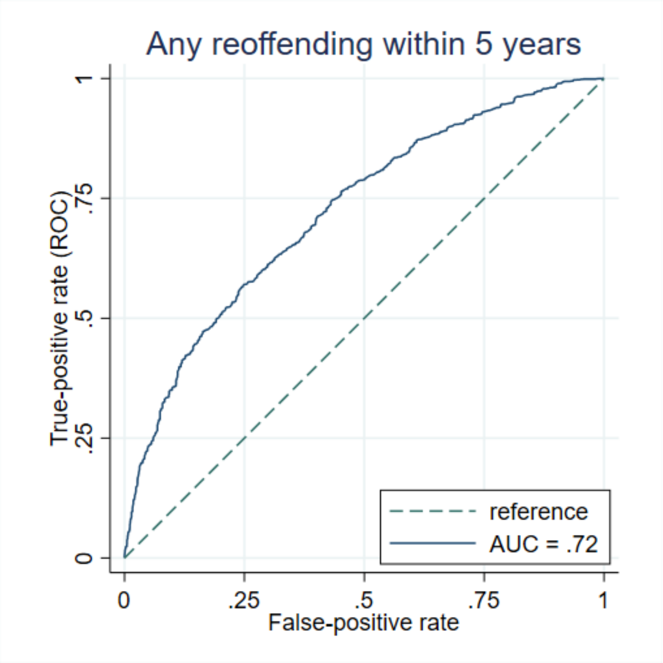

eFigure 5. Model discrimination shown by receiver operating characteristics curves for any conviction of a new crime

Note: AUC = areas under the receiver operating characteristics curve.

© 2023 Yu R et al. *JAMA Network Open.*

Derivation sample

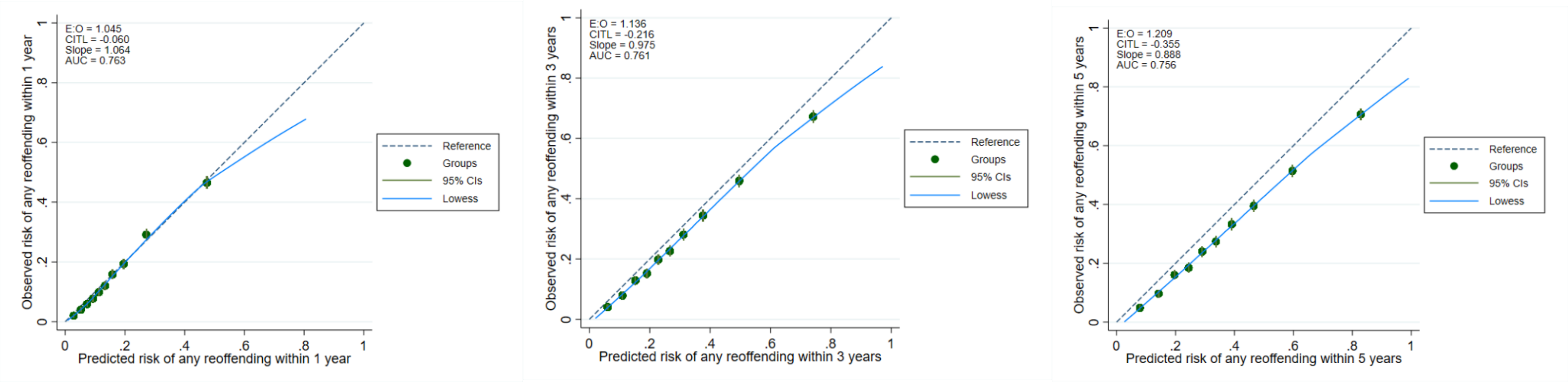

eFigure 6. Model calibration for any conviction of a new crime

© 2023 Yu R et al. *JAMA Network Open.*

eTable 6. Sensitivity, specificity, negative predictive value, and positive predictive value by different cut-off scores for probability of any reoffending

Cut-off	Sensitivity (95%CI)		Specificity (95%CI)		PPV (95%CI)		NPV (95%CI)		Accuracy (95%CI)	
Within 1 year (15.2%)										
5%	97.7	97.2, 98.2	16.2	15.7, 16.7	17.3	17.2, 17.4	97.5	96.9, 98.0	28.6	28.0, 29.2
10%	87.8	86.6, 88.9	43.5	42.8, 44.2	21.8	21.5, 22.1	95.2	94.8, 95.6	50.2	49.6, 50.9
15%	71.0	69.5, 72.5	68.0	67.3, 68.7	28.5	27.9, 29.1	92.9	92.5, 93.2	68.5	67.9, 69.1
20%	54.8	53.1, 56.5	81.6	81.1, 82.2	34.9	34.0, 35.9	91.0	90.6, 91.3	77.6	77.0, 78.1
Within 3 years (25.8%)										
5%	99.7	99.5, 99.8	4.4	4.1, 4.7	26.6	26.6, 26.7	97.7	96.3, 98.6	29.0	28.4, 29.6
10%	97.7	97.3, 98.1	16.4	15.8, 17.0	28.9	28.7, 29.1	95.3	94.5, 96.0	37.4	36.8, 38.0
15%	93.4	92.7, 94.0	30.5	29.8, 31.2	31.9	31.6, 32.1	93.0	92.3, 93.6	46.7	46.1, 47.4
20%	86.1	85.2, 87.0	45.5	44.8, 46.3	35.5	35.1, 35.9	90.4	89.8, 91.0	56.0	55.4, 56.7
Within 5 years (29.5%)										
5%	99.9	99.7, 99.9	2.1	1.9, 2.3	29.9	29.9, 30.0	97.0	94.6, 98.4	31.0	30.4, 31.6
10%	99.1	98.8, 99.3	10.3	9.8, 10.7	31.6	31.5, 31.8	96.5	95.5, 97.2	36.5	35.9, 37.1
15%	96.4	95.9, 96.9	21.9	21.2, 22.5	34.1	33.9, 34.3	93.6	92.8, 94.3	43.9	43.2, 44.5
20%	92.5	91.8, 93.1	33.2	32.4, 33.9	36.7	36.4, 37.0	91.3	90.6, 92.0	50.7	50.0, 51.3

Note: Data are given in percentages. 95%CI, 95% confidence interval; PPV, positive predictive value; NPV, negative predictive value.

eTable 7. Parameters to calculate predicted risk of domestic violence reoffending after arrest for domestic violence

Risk factors	Beta	LCI	UCI
Gender (male vs female)	.4716495	.218265	.725034
Unemployment	.1420303	.040481	.2435795
Alcohol use disorder	.1951887	.0750615	.3153159
Previous domestic violence	1.215765	1.044009	1.38752
Previous non-domestic violence	.5246828	.3769027	.672463
Previous non-violent offending	.2068696	.0407849	.3729544
Previous imprisonment	.3740933	.2490086	.499178

Note: LCI = Lower Confidence Interval; UCI = Upper Confidence Interval. Unemployment refers to status at time of arrest for domestic violence.

© 2023 Yu R et al. *JAMA Network Open.*

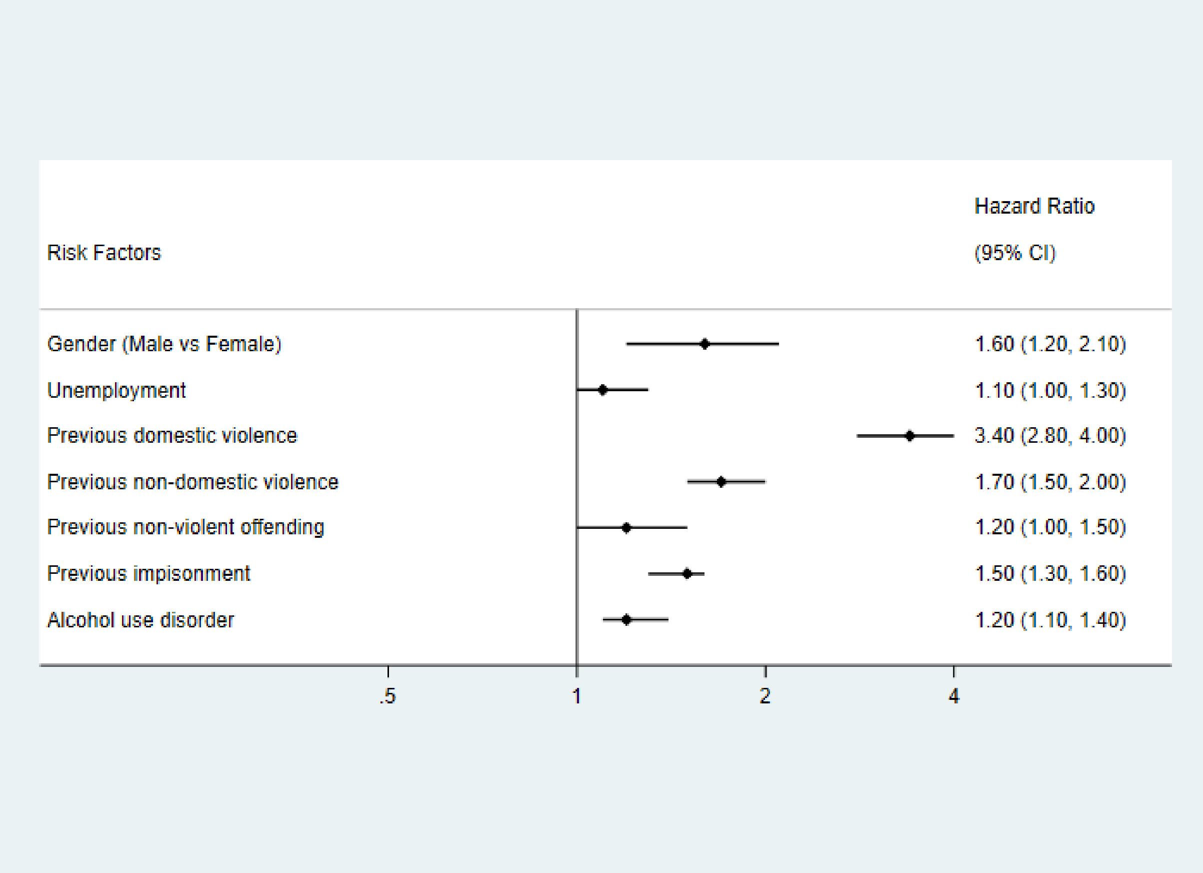

eFigure 7. Risk factors included in the final model for prediction of rearrest for domestic violence

Note: Unemployment refers to status at year of arrested for domestic violence. Hazard ratios were for the whole study period with a mean follow-up period of 25.7 months.

© 2023 Yu R et al. *JAMA Network Open.*

Derivation sample

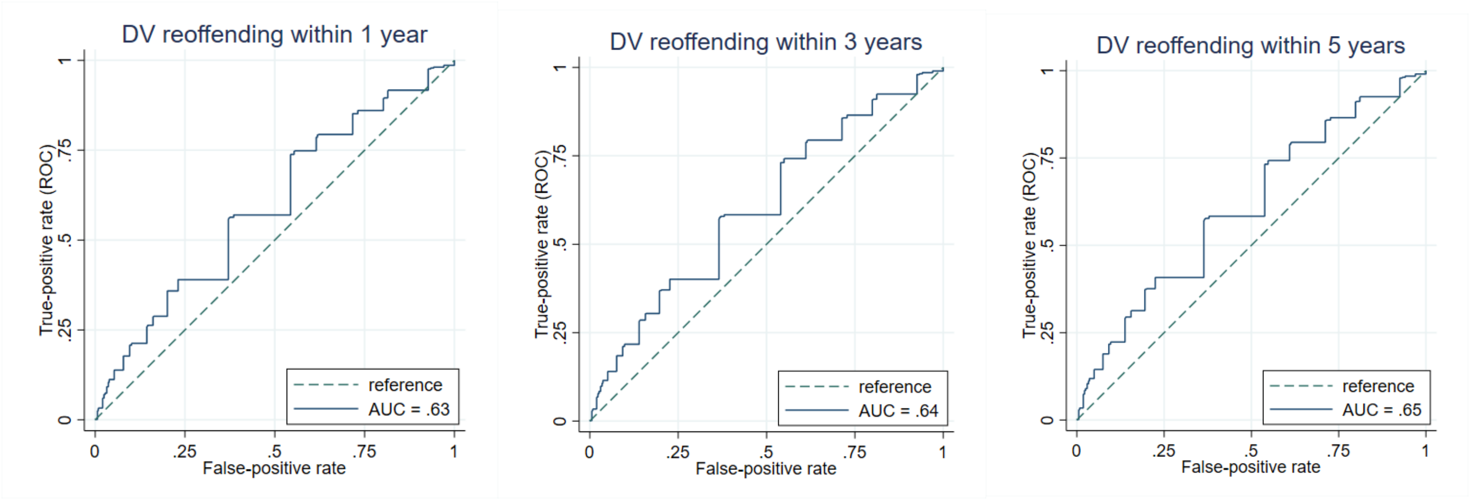

eFigure 8. Model discrimination shown by receiver operating characteristics curves for rearrest for domestic violence

Note: DV = Domestic Violence. AUC = areas under the receiver operating characteristics curve.

© 2023 Yu R et al. *JAMA Network Open.*

Derivation sample

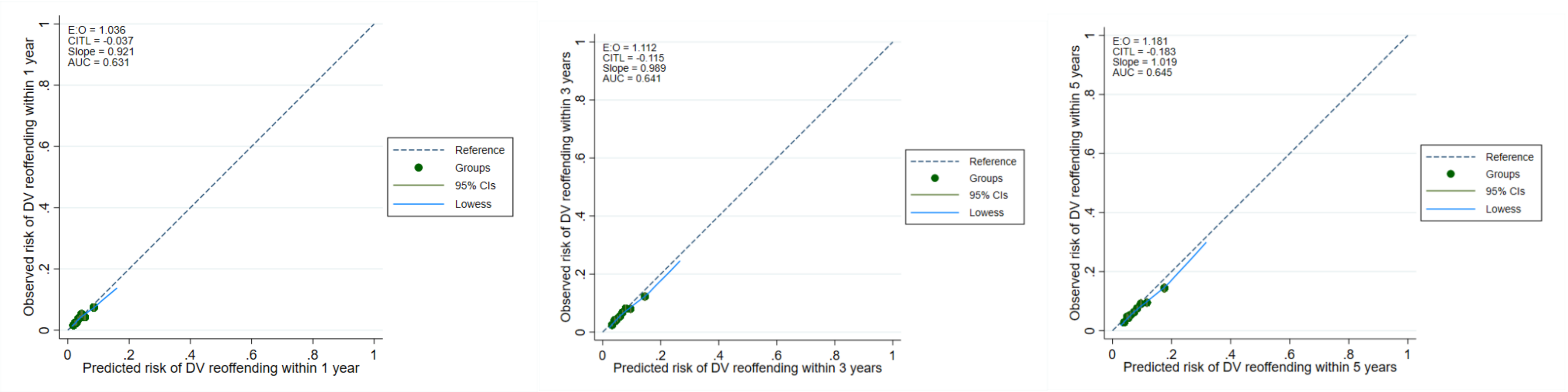

eFigure 9. Model calibration for domestic violence reoffending

Note: DV = Domestic Violence.

© 2023 Yu R et al. *JAMA Network Open.*

eTable 8. Sensitivity, specificity, negative predictive value, and positive predictive value by different cut-off scores for probability of domestic violence reoffending

Cut-off	Sensitivity (95%CI)		Specificity (95%CI)		PPV (95%CI)		NPV (95%CI)		Accuracy (95%CI)	
Within 1 year (3.6%)										
5%	26.3	23.3, 29.5	85.4	84.9, 85.9	6.3	5.6, 7.0	96.9	96.8, 97.0	83.3	82.8, 83.8
10%	5.9	4.4, 7.8	97.9	97.7, 98.1	9.5	7.3, 12.4	96.6	96.5, 96.6	94.6	94.3, 94.9
Within 3 years (5.8%)										
5%	79.3	77.0, 81.5	38.5	37.9, 39.2	7.4	7.2, 7.6	96.8	96.4, 97.1	40.9	40.3, 41.6
10%	21.7	19.5, 24.1	90.2	89.7, 90.6	7.6	6.8, 8.4	96.9	96.8, 97.0	87.7	87.3, 88.1
15%	9.6	8.3, 11.6	97.0	96.7, 97.2	10.7	9.1, 12.6	96.7	96.6, 96.	93.8	93.5, 94.1
20%	2.9	2.0, 3.9	99.4	99.3, 99.5	15.4	11.2, 20.7	96.5	96.5, 96.5	96.0	95.7, 96.2
Within 5 years (6.7%)										
5%	91.1	89.5, 92.5	20.2	19.6, 20.7	7.5	7.4, 7.6	97.0	96.4, 97.4	24.9	24.3, 25.5
10%	37.3	34.6, 39.8	80.5	80.0, 81.1	12.0	11.3, 12.8	94.8	94.5, 94.9	77.6	77.1, 78.2
15%	14.4	12.7, 16.3	95.0	94.7, 95.3	17.1	15.2, 19.1	94.0	93.9, 94.1	89.7	89.2, 90.1
20%	7.4	6.2, 8.9	98.1	97.9, 98.3	21.6	18.4, 25.3	93.7	93.6, 93.8	92.1	91.7, 92.4

Note: Data are given in percentages. 95%CI, 95% confidence interval; PPV, positive predictive value; NPV, negative predictive value. Accuracy: overall probability that a patient is correctly classified (=Sensitivity × Prevalence + Specificity × [1 – Prevalence]).

© 2023 Yu R et al. *JAMA Network Open.*
