# Supplementary material for: Development and Validation of a Prediction Tool for Reoffending Risk in Domestic Violence
Source: JAMA Netw Open. 2023 Jul 26;6(7):e2325494. doi: 10.1001/jamanetworkopen.2023.25494 (PMC10372708; doi:10.1001/jamanetworkopen.2023.25494)
Supplement: Supplement 2. — Data Sharing Statement [file jamanetwopen-e2325494-s002.pdf]

Data Sharing Statement

Yu. Development and Validation of a Prediction Tool for Reoffending Risk in Domestic Violence. *JAMA Netw Open*. Published July 26, 2023.
doi:10.1001/jamanetworkopen.2023.25494

Data

Data available: The Public Access to Information and Secrecy Act in Sweden prohibits us from making individual level data publicly available. Researchers who are interested in replicating our work can apply for individual level data from: Statistics Sweden (mikrodata@scb.se) for data from The Total Population Register (https://www.scb.se/vara-tjanster/bestallamikrodata/vilka-mikrodata-finns/individregister/ registret-over-totalbefolkningen-rtb/), The MultiGeneration Register (https://www.scb.se/varatjanster/bestalla-mikrodata/vilka-mikrodata-finns/ individregister/flergenerationsregistret/), The Longitudinal Integrated Database for Health Insurance and Labour Market Studies (https:// www.scb.se/en/services/guidance-for-researchersand-universities/vilka-mikrodata-finns/ longitudinella-register/longitudinal-integrateddatabase-for-health-insurance-and-labour-marketstudies-lisa/), The National Board of Health and Welfare (registerservice@socialstyrelsen.se) for data from The Patient Register (https://www. socialstyrelsen.se/patientregistret), and The Swedish National Council for Crime Prevention (statistik@bra.se) for data from The National Crime Register (https://www.bra.se/statistik/ kriminalstatistik/specialbestallningar.html).
